# Supplementary material for: Coupling coordination between environment, economy and tourism: A case study of China
Source: PLoS One. 2020 Feb 4;15(2):e0228426. doi: 10.1371/journal.pone.0228426 (PMC6999866; doi:10.1371/journal.pone.0228426)
Supplement: Attachment — Submitted filename: Response to Reviewers.docx [file pone.0228426.s006.docx]

Response to Reviewers

Q1. I think that the authors have not adequately described their analysis in this original manuscript, for example, how the authors obtained datasets, any parameters used, and please keep in mind that, if any existing datasets were used for testing, it should have enough details for another researcher to reproduce the findings. A data-source should be presented in the context or as a supp. file.

**Answer:** We made a detailed description of the selection of data sources and indicators in the revised version, and added the datasets as an attachment to the revised version.

Q2. References are not sufficient. At least, please add some relevant references in the first Paragraph of the introduction section. And a literature review is suggested to be added.

**Answer:** We have added references and literature reviews to the introduction.

Q3. The language should be improved.

**Answer:** We have employed the AJE (<http://learn.aje.com>) to thoroughly copyedit this manuscript for language usage, spelling, and grammar.

Q4. Policy implications should be considered as a solid section before the conclusions.

**Answer:** We rewrote the policy recommendations and placed them separately in Section 3.4.

Q5. Please try your best to concentrate your findings from the modelling analysis, and add some discussions by such as comparative analysis with the external outcomes from other scholars.

**Answer:** We have added some comparative discussions with other literature results in the empirical analysis section.
