# Supplementary material for: Coupling coordination between environment, economy and tourism: A case study of China
Source: PLoS One. 2020 Feb 4;15(2):e0228426. doi: 10.1371/journal.pone.0228426 (PMC6999866; doi:10.1371/journal.pone.0228426)
Supplement: Attachment — Submitted filename: Comments to PONE-D-19-27598_R1.docx [file pone.0228426.s007.docx]

This manuscript adopted a coupling coordination model to analyze the relationship between ecological environment, economy and tourism in China’s provinces during 2003-2017. However, it could not be published in its current form.

First, the research contributions are not clear. A potential innovation in this manuscript is its consideration of using coupling model to analysis the relationship between environment, economy and tourism within China. previous papers have included this development and its application in understanding China’s coordination development between economy and tourism and environment. If there is no innovation in the method, what is your most important contribution?

Second, you can refer to some more professional research to standardize the expression, for instance, coupling coordination degree or coordination degree? Tourism is an integral part of the economy, thus, Is it feasible to use “coupling” to describe the relationship between tourism industry and economy.

Third, the manuscript contains statements that are too hard to understand. It's almost like the author(s) had run out of steam at this stage and as the reader, I must say that I felt relieved to have completed the marathon! Specific comments with regard to grammar, flow and basic expression are too numerous to mention. Assistance with writing skills and grammar is needed through an independent third party - someone who knows little about the technical content but can assist with these shortcomings. The paper falls short of being a professionally written management article.

Four, It needs a major rewrite and rethink to eliminate redundant material and unrelated debate and discussion. For example, the sentence in lines 28-30, page 1; the last sentence in 3.4, page 15, etc.

The results are not clearly presented, for example, especial spatial distribution pattern of coupling coordination development for the 31 provinces.

Some technical erros: “they have good representativeness, understanding, availability, integrity and dissemination”(lines 3-4, page 4), pay attention to “understanding”

“are negative indexes (the larger the better), the other indexes are all positive indicators (the smaller the better)”((lines 3-4, page 4), for positive indicators, It should be “the larger the better”

Section 3.4 contains statements that are just far too basic and obvious. Policy recommendations are not targeted, they should be given on the basis of your results specifically.
