# Supplementary material for: Coupling coordination between environment, economy and tourism: A case study of China
Source: PLoS One. 2020 Feb 4;15(2):e0228426. doi: 10.1371/journal.pone.0228426 (PMC6999866; doi:10.1371/journal.pone.0228426)
Supplement: Attachment — Submitted filename: Response to Reviewers.docx [file pone.0228426.s008.docx]

Dear Reviewers:

Thank you for your comments concerning our manuscript entitled “ Coupling coordination between environment, economy and tourism：A case study of China” (No.: PONE-D-19-27598R1). Those comments are all valuable and very helpful for revising and improving our paper. We have studied comments carefully and have made correction which we hope meet with approval. The main corrections in the paper and the responds to the reviewer’s comments are as follows.

Responds to the reviewer’s comments:

Reviewer #1:

*1.The introduction part is not sufficient. Especially, the contribution is unclear enough. Hence, the authors should rewrite it.*

Response: Thanks for your suggestions. We have rewritten the introduction in the ‘Manuscript’ (Lines 27-85).

*2.Table 1 needs to be improved. For Table 2,3, and 4, the authors had better convert them to figures in a bid to improve the readability.*

Response: Thanks for your suggestions. We have improved the Table 1 in the ‘Manuscript’. The Table 2, 3, and 4 have been converted to the Figure 2, 3, and 4 in the ‘Manuscript’ (Lines 286-288, 345-347, 401-402).

*3. Policy recommendations should be moved to the end. Besides, it should be rewritten based on the conclusions.*

Response: Thanks for your suggestions. We have rewritten the policy recommendations based on the conclusions and moved the policy recommendations to the end in the ‘Manuscript’ (Lines 435-451).

Reviewer #2:

1. *the research contributions are not clear. A potential innovation in this manuscript is its consideration of using coupling model to analysis the relationship between environment, economy and tourism within China. previous papers have included this development and its application in understanding China’s coordination development between economy and tourism and environment. If there is no innovation in the method, what is your most important contribution?*

Response: Thanks for your suggestions. We are sorry that the innovations of the paper was not clear in the original manuscript. The innovations of the paper includes two parts:1)Previous studies mainly focused on single province in China, the studies based on large scale are lack; 2)there are fewer studies to analyze the spatial changes of China’s coordination development between environment and economy and tourism, and the paper makes up for this gap (Lines 72-85).

*2. you can refer to some more professional research to standardize the expression, for instance, coupling coordination degree or coordination degree? Tourism is an integral part of the economy, thus, Is it feasible to use “coupling” to describe the relationship between tourism industry and economy.*

Response: Thanks for your suggestions. We refer to the references by Sheng et al.(2009), Yuan et al.(2014) and Zhou et al.(2016a, 2016b, 2016c), all of which use coupling coordination to study the relationship between tourism industry and the economy. We believe that it is feasible to use “coupling coordination” to describe the relationship between tourism industry and economy.

These references are as follows:

[1] Sheng Y C, Zhong Z P. Study on the Coupling Coordinative Degree between Tourism Industry and Regional Economy: A Case Study of Hunan Province. Tourism Tribune. 2009; 24(8): 23-29.

[2] Yuan Y, Jin M, Ren J, Hu M, Ren P. The dynamic coordinated development of a regional environment-tourism-economy system: A case study from western Hunan province, China. Sustainability. 2014; 6(8): 5231-5251. https://doi.org/10.3390/su6085231

[3] Zhou C, Feng X G, Tang R. Analysis and forecast of coupling coordination development among the regional economy-ecological environment-tourism industry: A case study of provinces along the Yangtze economic zone. Economic Geography. 2016a; 36: 186-193.

[4] Zhou C, Feng X G, Tang R. Analysis and forecast of coupling coordination development among the regional economy-ecological environment-tourism industry - A case study of provinces along the Yangtze Economic Zone. Economic Geography. 2016b; 36(3): 186–193.

[5] Zhou C, Jin C, Zhao B, Zhang F. The provincial difference of coupling coordinative development of regional economy-ecology-tourism. Journal of Arid Land Resources and Environment. 2016c; 30(7):203-208.

*3. the manuscript contains statements that are too hard to understand. It's almost like the author(s) had run out of steam at this stage and as the reader, I must say that I felt relieved to have completed the marathon! Specific comments with regard to grammar, flow and basic expression are too numerous to mention. Assistance with writing skills and grammar is needed through an independent third party - someone who knows little about the technical content but can assist with these shortcomings. The paper falls short of being a professionally written management article.*

Response: Thanks for your suggestions. This manuscript has been revised in the English format. Before we submitted the original manuscript, Dr. Yan Dan has helped us revise the English format. She received her doctorate degree from Wageningen University, and her English level reached the native language level. Before we submitted the manuscript, we employed American Journal Experts (AJE) to revise the English format. The editing certificate is as follows.

*4. It needs a major rewrite and rethink to eliminate redundant material and unrelated debate and discussion. For example, the sentence in lines 28-30, page 1; the last sentence in 3.4, page 15, etc.*

Response: Thanks for your suggestions. We have rewritten the introduction in the ‘Manuscript’ (Lines 27-85). And we have rewritten the policy recommendations based on the conclusions and moved the policy recommendations to the end in the ‘Manuscript’ (Lines 435-451).

*5.The results are not clearly presented, for example, especial spatial distribution pattern of coupling coordination development for the 31 provinces.*

Response: Thanks for your suggestions. We have converted The Table 2,3, and 4 to the Figure 2,3, and 4 in the ‘Manuscript’(Lines 286-288, 345-347, 401-402) and analyzed the spatial distribution pattern of coupling coordination development for the 31 provinces.

*6.Some technical erros: “they have good representativeness, understanding, availability, integrity and dissemination”(lines 3-4, page 4), pay attention to “understanding” “are negative indexes (the larger the better), the other indexes are all positive indicators (the smaller the better)”((lines 3-4, page 4), for positive indicators, It should be “the larger the better”*

Response: Thank you very much for reviewers’ suggestions. We have corrected the sentence in the ‘manuscript’ (Lines 97 & 98).

*7.Section 3.4 contains statements that are just far too basic and obvious. Policy recommendations are not targeted, they should be given on the basis of your results specifically.*

Response: Thanks for your suggestions. We have rewritten the policy recommendations based on the conclusions and moved the policy recommendations to the end in the ‘Manuscript’ (Lines 435-451).

Reviewer #3:

*1.Line 7 of the first Paragraph in Section 1: “The impact and pressure of this extensive economic development has (should be have) led to…”. There exists an inappropriate tense.*

Response: Thanks for your suggestions. We have rewritten the introduction in the ‘Manuscript’ (Lines 27-85).

*2.Line 1 of the second Paragraph in Section 1: “…, much literature has studied the interaction …”. There exists an inappropriate tense.*

Response: Thanks for your suggestions. We have corrected the sentence in the ‘manuscript’ (Lines 46-47).

*3. Lines 1, 2 & 3 of the last Paragraph in Section 1: “…, whether the ecological environment can be coordinated with economic and tourism development has become a problem that many scholars and government departments need to study.”. The narrative here should be improved.*

Response: Thanks for your suggestions. We have rewritten the introduction in the ‘Manuscript’ (Lines 27-85).

*4. The first Section of the paper does not significantly explain what contributions it has made. It is suggested that the main contributions of the paper be listed in the last Paragraph of Section 1.*

Response: Thanks for your suggestions. We are sorry that the main contributions of the paper have not been clearly expressed the main contributions in the original manuscript. The innovations of the paper includes two parts:1)Previous studies mainly focused on single province in China, the studies based on large scale are lack; 2)there are fewer studies to analyze the spatial changes of China’s coordination development between environment and economy and tourism, and the paper makes up for this gap (Lines 72-85).

*5. The entropy weighting results of the three subsystems in Section 2.4 are not the focus of the paper. It is recommended to replace Table 1 with a concise language narrative.*

Response: Thanks for your suggestions. We have revised the Section 2.4 and replaced Table 1 with a concise language narrative (Lines 205-207).

*6. The results in Sections 3.2 and 3.3 are relatively thorough, but the results expressed in tabular form are worse than the graphical representation. It is recommended to replace Tables 2, 3 and 4 with the corresponding graphical representation.*

Response: Thanks for your suggestions. We have converted The Table 2,3, and 4 to the Figure 2,3, and 4 in the ‘Manuscript’(Lines 286-288, 345-347, 401-402) and analyzed the spatial distribution pattern of coupling coordination development for the 31 provinces.

*7. The conclusions of Section 4 are too much and too long. It is recommended to reduce the length and express the main conclusions of the paper in a clear and concise language.*

Response: Thanks for your suggestions. We have revised the ‘Conclusion and policy recommendations’ in the ‘Manuscript’ (Lines 404-451).

At last, we tried our best to improve the manuscript and made some changes in the manuscript. We really appreciate it that Reviewers help up improve the quality of the research, and hope that the correction will meet with approval. Once again, thank you very much for your comments and suggestions.

Yours Sincerely

Zhizhu Lai and Zheng Wang
